# A “sandwich” strategy for functional cure of chronic hepatitis B

**DOI:** 10.1038/s41426-018-0092-3

**Published:** 2018-05-17

**Authors:** Xuan-Yi Wang, Yu-Mei Wen

**Affiliations:** 1 Key Laboratory Medical Molecular Virology, MoE/MoH, Shanghai Medical College, Fudan University, Shanghai, China 200032; 2 Institutes of Biomedical Sciences, Shanghai Medical College, Fudan University, Shanghai, China 200032

Dear Editor,

Hepatitis B virus (HBV) belongs to the ortho-hepadnaviridae, with a partial circular DNA genome, encoding the surface protein (HBsAg), core protein (HBcAg), X protein (HBxAg), and HBV polymerase. HBV replicates via an RNA intermediates and forms a covalently closed circular DNA (cccDNA) in cellular nuclei, which can be replenished to persistently express viral proteins and replication. Uniquely, the small spherical HBsAg particles transcribed by the viral genome reside in the sera of HBV-infected individuals can reach a number of as high as 10^12–14^/ml. Furthermore, HBsAg is the major protein that antagonizes various host immune mechanisms, which is one of the major mechanisms for HBV persistence in hosts.

Despite the remarkable success of hepatitis B prophylactic vaccination program in many countries, there are still more than 240 million people worldwide chronically infected with HBV, and up to one million deaths every year is caused by HBV-related cancer or end stage liver diseases^1^. Therefore, chronic hepatitis B (CHB) is of global concern. Currently, only anti-HBV nucleosides (nucleotides) drugs and interferon-α (IFN-α) are licensed for treatment of the CHB patients. The anti-HBV drugs only target the reverse transcriptase domain of HBV polymerase, which can effectively inhibit the replication of HBV, and decrease the viral load, but these drugs have no inhibitory effects on HBV cccDNA. Similarly, IFN-α only nonspecifically inhibits viral replication and regulates certain immune response, and has no effects on cccDNA either. Since the current treatment cannot eliminate HBV or cure HBV infection, life-long treatment with antiviral or IFN-α is inevitable, with the risk of developing drug resistance or severe side effects.

As the goal of complete elimination of HBV in CHB patients is difficult to attain, in recent years, a consensus has been reached aiming at “functional cure” for the CHB patients. The definition of functional cure includes durable HBsAg loss (with or without HBsAg sero-conversion), undetectable serum HBV DNA, persistence of cccDNA in a transcriptionally inactive status, and the absence of spontaneous relapse after the cessation of treatment^2^. Previously, two interrelated arms of the CHB therapies, namely antiviral treatment and immunotherapies, have been explored and some are under clinical trials. To achieve functional cure, these two approaches should be upgraded, by which, antiviral treatment should be effective in both inhibiting HBV replication and decreasing serum HBsAg, while immune therapy should restore adaptive immune responses versus HBV to provide long-term immune control of HBV against spontaneous relapse after cessation of treatment.

Recently, several reports observed that by either “early switch to” or “late add-on” combination of antiviral drugs with peg-IFN showed additive effects to certain extent^3^. When patients under long-term antiviral drug treatment resulted in low-level of serum HBsAg (<3 log of serum HBsAg), clearance of HBsAg was observed in some patients, when they further received peg-IFN treatment^4, 5^. These findings seemed to be due to IFN’s effects on cccDNA in HBV-infected cells. Interferon has been shown to trigger non-cytolytic degradation of cccDNA in infected cells, and activation of nuclear deaminases, resulted in cccDNA deamination leading to a significant reduction of cccDNA^6^. These observations provided clues to employ different immune therapies in patients with low levels of HBsAg, and several clinical trials are undergoing^7^. In addition, human anti-HBs and anti-pre-S1-monoclonal antibodies have been developed recently and have shown to clear serum HBsAg in different mouse models^8–10^. These studies provide renewed interest of employing neutralizing antibodies as a therapeutic approach against serum HBsAg.

**Fig. 1:**
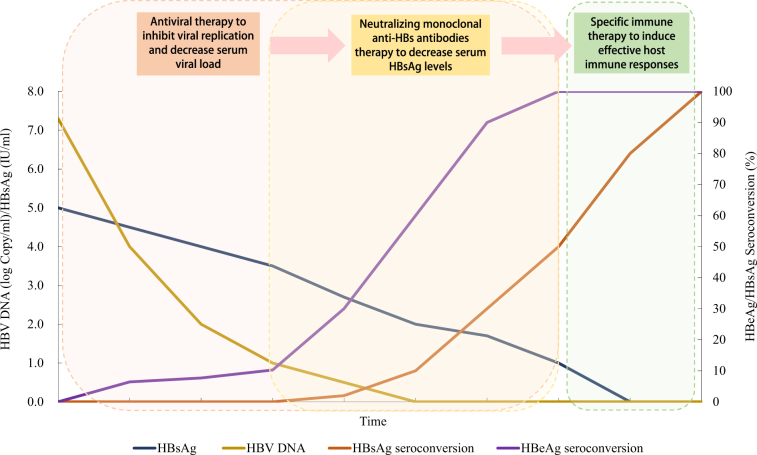
Diagram of expected therapeutic efficacy under proposed “sandwich” strategy

As only few CHB patients under long-term antiviral treatment can reach a significant decrease in serum HBsAg. Of these, only a part of the individuals with further treatment may reach functional cure. While human neutralizing anti-HBs/anti-Pre S1 antibodies may help to decrease the load of serum HBsAg, we herein propose a “sandwich” approach to expedite the decrease of serum HBsAg in the CHB patients, and to induce potent-specific immune responses to prevent spontaneous relapse after the cessation of treatment. This approach consists of the following protocols: (1) use antiviral drugs to inhibit viral replication and decrease serum viral load, throughout the whole therapeutic process as the first layer of sandwich; (2) employ potent neutralizing monoclonal anti-HBs antibodies to decrease serum HBsAg levels, mimicking the decrease of HBsAg after long-term antiviral therapy as the second layer of sandwich; and, (3) when patients were free from serum HBV DNA and HBsAg, with a transient “window stage” similar to normal adults, potent-specific active immunization should be applied to induce effective host immune responses serving as the last layer. A diagram of the most optimistic expected therapeutic efficacy of this strategy is shown in Figure 1.

The uniqueness of this approach is by two combinations, one is the combination between antiviral drugs and immunotherapy, the other is by combination of passive and active immunization. Neutralizing anti-HBs human antibodies, can neutralize HBV via the Fab fragment, while the Fc fragment can further enhance host immune response via multiple mechanisms, such as ADCC, etc.^11, 12^. A cocktail of anti-HBs and anti-pre-S1-monoclonal antibodies may jointly block the entry of HBV to infect new hepatocytes, and restore damaged host immune responses exerted by high levels of HBsAg^10^. So far, though active immunization by therapeutic vaccination in CHB patients showed limited efficacy, restoration of CD4^+^ and CD8^+^ cell functions, decrease of Treg cells, and effective HBeAg sero-conversion, as well as one-log decrease of serum HBsAg have been observed in clinical trials of HBsAg-HBIG immune complex therapeutic vaccine^13, 14^. Furthermore, very low levels of B cells against HBsAg have been observed in the CHB patients^15^. These cells, probably inactivated by the persistence of HBsAg, may restore their active functions via potent active immunization, and help to prevent spontaneous relapse after cessation of treatment.

Though this “sandwich” approach seems applicable, the short “window stage” of transient clearance of serum HBsAg is critical for successful treatment of CHB patients. “Add on” or different sequential protocols need to be explored to make use of this stage, and potent active immunization should be explored. To avoid possible side effects, liver functions, humoral, and cellular immune responses should be closely monitored during this protocol of treatment. In addition, different active immunization regimens, such as, DNA vaccination, vector-mediated vaccines, usage of different adjuvants, etc., are all possible approaches that should be studied to maximize the desired anti-HBV immune responses. As all approaches for CHB functional cure can only be verified by clinical trials, we hope the approach presented in this article can be considered for international collaboration, and granted approval for pilot clinical trials in the near future, to benefit patients suffering from the consequences of CHB.
